# A New Seamless Transfer Control Strategy of the Microgrid

**DOI:** 10.1155/2014/391945

**Published:** 2014-05-22

**Authors:** Zhaoyun Zhang, Wei Chen, Zhe Zhang

**Affiliations:** State Key Laboratory of Advanced Electromagnetic Engineering and Technology, Huazhong University of Science & Technology, Wuhan 430074, China

## Abstract

A microgrid may operate under two typical modes; the seamless transfer control of the microgrid is very important. The mode conversion controller is installed in microgrid and the control logic of master power is optimized for microgrid mode conversion. In the proposed scheme, master power is very important. The master-power is under the PQ control when microgrid is under grid-connected. And it is under V/F control when the microgrid is under islanding. The microgrid mode controller is used to solve the planned conversion. Three types of conversion are simulated in this paper. The simulation results show the correctness and validity of the mode control scheme. Finally, the implementation and application of the operation and control device are described.

## 1. Introduction

A microgrid is a low-voltage distribution grid comprising various controllable loads, storage devices, and distributed generators as a controlled entity that can either be isolated from or operate interconnectedly with the main grid. Distributed generation (DG) and the microgrid (MG) system have received increasing research attention [1–6]. At the same time, many demonstration projects of microgrids have been constructed in China, such as Dongfushan Island microgrid in Zhejiang and Zhangbei microgrid in Hebei. In general, demonstration projects involve the power such as photovoltaic, wind, and storage battery [7–9].

The microgrid may operate under two typical modes: it can connect with the main grid, known as grid-connected mode (GM), and it can operate without main grid, called islanding mode (IM). Mode conversion is one of the core issues of the microgrid control. The researches have focused on the grid-connected mode inverter control [10–12], but few research has been done on the mode conversion.

The microgrid control may be implemented under the master-slave control mode, droop control mode [13, 14], and so forth. However, many microgrids have been built or under construction adopting master-slave mode, mainly because this type of microgrids can keep the voltage and frequency of the microgrid near nominal point. On the other hand, the active power of the solar and wind energy usually is not controllable continuously, and PV systems and wind turbines often work at the maximum power point (MPP). So, control of solar and wind energy is in PQ control mode whether it is under islanding mode or grid-connected mode. Control logic is relatively simple. The reactive and active power of energy storage can be adjusted, and the energy storage system becomes the “master” power of microgrid when the microgrid is under islanding mode, and it is the frequency and voltage support of microgrid.

Frequency and voltage of the “master-slave” architecture microgrid can remain near the nominal point, control structures is clear, the control logic of the “slave” power is simple, and the “slave” power has the plug and play features. Because of these advantages, this microgrid architecture has been used in a wide range of applications. In papers [15–17], dual-mode inverter for this type of microgrid was researched, and the system is under PQ control when grid-connected mode is adopted; the system is under V/F control when islanding mode is adopted. The authors also proposed a microgrid mode conversion as a preliminary study but did not give more specific solutions.

The mode conversion of microgrid with “master-slave” architecture is discussed in this paper. The microgrid mode conversion includes the following four types:

1. planned conversion from grid-connected mode to islanding mode,
2. unplanned conversion from grid-connected mode to islanding mode,
3. planned conversion from islanding mode to grid-connected mode,
4. unplanned conversion from islanding mode to grid-connected mode.

The fourth conversion can be avoided, but others cannot. In this study, for the microgrid mode conversion, microgrid sets a centralized mode controller and optimizes the master-power's control logic. The unplanned mode conversion is solved by the logic optimization of the master power, and the planned mode conversion is solved by the mode controller and the logic optimization of the master power.

A detailed program of the mode controller and an optimization scheme of the master power converter control system are presented. The energy storage system as an example of master power is described. The master power operates under PQ mode when microgrid works under grid-connected mode and master power operates under V/F mode when microgrid operates under islanding mode. The microgrid operating mode is detected through the microgrid information such as current, voltage, and digital input. The master power will change the operating mode, when the microgrid changes its operating mode. In the mode conversion process, a series of programs will be used to ensure microgrid stability.

## 2. Design of Control System

In a large number of the latest microgrid demonstration projects, a microgrid includes the photovoltaic power generation system, wind power systems, and energy storage system in which the “wind-solar-storage” mode is adopted. There are the photovoltaic power generation system, wind power systems, and energy storage system. Therefore, this study focuses on this type of microgrids. Without loss of generality, all of the PV systems are equivalent to one photovoltaic power generation system; all the wind systems in parallel are equivalent to one wind power system; all of the energy storage systems in parallel are equivalent to one storage system; load is distributed in microgrid.

In photovoltaic systems and direct drive wind power generation system of the microgrid, which are under PQ control mode, the maximum power tracking is always used and reactive power output is always 0.

Storage system is under the PQ control when microgrid is under grid-connected mode. The system is under V/F control, when the microgrid is under islanding.

To achieve the smooth transition between islanding mode and grid-connected mode, the control of the microgrid mode conversion includes two parts: one is the conversion control system between PQ control and V/F control for the storage system and the other is the mode controller for the mode conversion of microgrid.

### 2.1. Mode Conversion for the Storage System

The mode conversion between the PQ control and the V/F control for the storage system is a key component for the mode conversion of the microgrid.

#### 2.1.1. PQ Control of the Storage System

**Figure 1:**
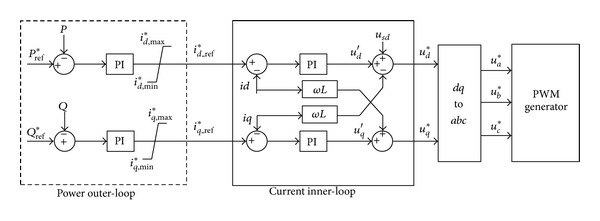
Schematic diagram of the PQ controller.

When the storage system is under PQ control mode, the double loop control is used, which includes power outer-loop control and current inner-loop control.  Figure 1 is the control diagram.

In power outer loop, the energy management system of the microgrid provides the active power reference value *P*_ref_* and reactive power reference value *Q*_ref_*, which depend on the state of the storage system and the load balance of microgrid.

The difference between the reference value and the actual value of active power is the input of the outer-loop PI regulator, for which the output is the reference values of the *d*-axis current *i*_d_* in the inner loop. Value of the *q*-axis current *i*_q_* is similar to *i*_d_*.

In current inner loop, the difference between reference value and actual value of the *d*-axis current is the input of the PI regulator, for which the output is the reference value of the *d*-axis voltage (*u*_d_*) of the inverter. The difference between reference value and actual value of the *q*-axis current is the input of the PI regulator, for which the output is the reference value of the *d*-axis voltage (*u*_q_*) of the inverter.

#### 2.1.2. V/F Control of Storage System

**Figure 2:**
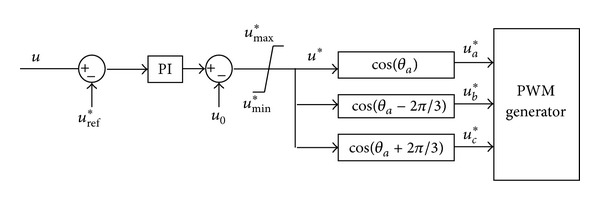
Schematic diagram of the V/F controller.

When storage system is under V/F control mode, the voltage reference value is set. And frequency reference value is set, too. Voltage difference between reference value and the actual value is the input of PI regulator, for which the output is the reference values of the control voltage of the inverter.  Figure 2 is the control diagram.

Where, *u* is the actual value of the voltage of the storage system output, u* is the reference of microgrid voltage, *f* is the microgrid frequency, which can be set at 50 Hz (or 60 Hz), *θ*_0_ is the initial phase angle, and *u*_0_ is the initial voltage value.

To keep frequency and voltage of the microgrid stable, angle *θ*_0_ and voltage *u*_0_ will be adjusted.

#### 2.1.3. Mode Conversion of the Storage System

Whether the system is under PQ control mode or V/F control mode, the last step is to get *u*_a_*, *u*_b_*, and *u*_c_* to PWM driver circuit and control IGBT turn-off and turn-on. In order to ensure a smooth transition, the reference voltages *u*_a_*, *u*_b_*, and *u*_c_* must be changed smoothly.

To ensure the continuity of the PWM reference voltage, it is necessary to ensure continuous amplitude and phase.

When the microgrid switches from grid-connected mode to islanding mode, storage system switch control includes the following.

(a) The amplitude *u*_a_* and phase angle *θ*_0_ of PWM reference voltage are recorded when system is under grid-connected mode.
(b) When the microgrid switches from grid-connected mode to islanding mode, storage system is from the PQ control mode to the V/F control mode. The voltage reference value *u*_0_ in V/F control mode is equal to the recording amplitude, when system is under PQ control mode, and the phase angle is
  (1) $$\begin{array}{c} \theta_{a}=\theta_{0}+2\pi f\Delta t, \end{array}$$

where *θ*_0_ is the recording phase angle when the storage system is under PQ mode, Δ*t* is time after the storage system switches into the V/F control mode, and *f* is the voltage frequency value of the microgrid.

When microgrid operates continuously under V/F mode, the recursive algorithm is used in the phase angle calculation; the formula is

(2) $$\begin{array}{c} \theta_{t}=\theta_{t-\tau }+2\pi f\tau , \end{array}$$

where *θ*_*t*_ is the phase angle of the current sampling point, *θ*_*t*−*τ*_ is the phase angle of the latest sampling point, *τ* is the sampling interval, and *f* is frequency value of microgrid.

When microgrid switches from islanding mode to grid-connected mode, the synchronization function is fulfilled by the mode controller of microgrid. The storage system only needs to change the response time of the PQ control mode (increasing *K*_*i*_ and reducing *K*_*p*_) and the system can complete the smooth transition from the V/F control mode to the PQ control mode.

#### 2.1.4. Inverter Parameters

The storage system inverter control includes the following parameters:

- the control mode: the PQ mode or V/F mode, which can through an external input, active detection, or the set of the inverter,
- the active power reference value: valid under PQ mode,
- the reactive power reference value: valid under the PQ mode,
- the voltage reference value: valid under V/F mode,
- the frequency reference value: valid under V/F mode,
- the phase angle reference values: valid under V/F mode; this generally is not for the external input, instead of the internal automatic continuous calculation.

### 2.2. The Mode Controller of Microgrid

With mode conversion of storage system, microgrid can smoothly switch between grid-connected mode and islanding mode. The microgrid mode controller is used to solve two problems and accomplish two goals as follows:

- planned conversion from grid-connected mode to islanding mode,
- planned conversion from islanding mode to grid-connected mode.

#### 2.2.1. Planned Conversion from Grid-Connected Mode to Islanding Mode

Planned conversion from grid-connected mode to islanding mode does not trip the PCC's breaker immediately. A series of control methods are adopted via the mode controller of the microgrid, and the appropriate time for the trip breaker is selected, and then the system switches from the grid-connected mode to the islanding mode.

Control logic is as follows.

**Figure 3:**
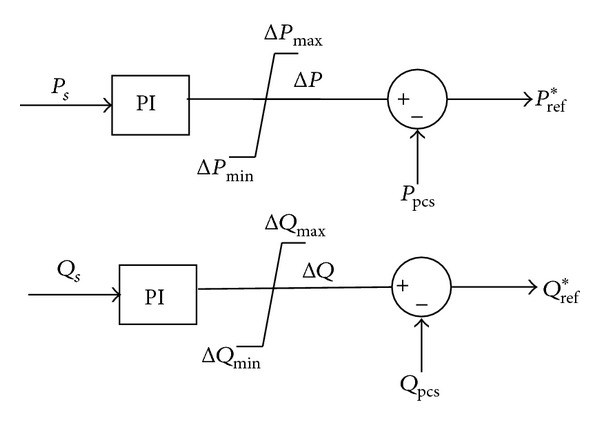
Schematic diagram of the power adjustment.

(a) After splitting command is produced, the storage system continues to work under PQ control mode. The mode controller begins to change the reference power of the storage system until the apparent power reaches 0. The control logic is as shown in Figure 3.

In Figure 3, *P*_*s*_ is the output active power from microgrid to main grid, *Q*_*s*_ is the output reactive power from microgrid to main grid, *P*_pcs_ is the actual output active power of PCS (storage system), *Q*_pcs_ is the actual output reactive power of PCS, *P*_ref_* is reference value of output active power of PCS, and *Q*_ref_* is reference value of output reactive power of PCS.

(b) If the active power and reactive power of microgrid are lower than the given values, the microgrid mode controller trips PCC breaker.

The microgrid mode controller monitors apparent power amplitude *S*_*s*_ = *P*_*s*_ + *jQ*_*s*_ in real time. When the amplitude is continuously lower than the threshold value for a period of time (e.g., 0.1 s), the mode controller of the microgrid sends the command to trip the PCC breaker. The storage system continues to work under PQ control mode.

(c) After the trip command is received, PCC breaker turns off, and the microgrid works under the islanding mode. The storage system detects the islanding mode and starts to work under V/F control mode. Planned conversion is completed.

#### 2.2.2. Planned Conversion from Islanding Mode to Grid-Connected Mode

With planned conversion from islanding mode to grid-connected mode, the synchronization problem between microgrid and main grid is resolved. Frequency and voltage adjustment is adjusted to allow microgrid to eventually meet the synchrony conditions.

**Figure 4:**
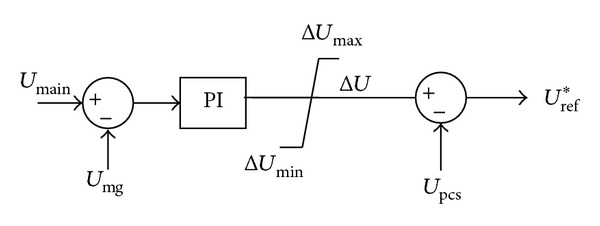
The voltage amplitude adjustment diagram.

The control logic is as follows:

 (a) voltage adjustment: through adjusting the voltage reference value of storage system, the microgrid voltage becomes closer to main grid's voltage; the control logic is shown in Figure 4;
 (b) frequency adjustment: the mode controller detects the main grid's frequency and phase difference between the microgrid and main grid; then microgrid frequency is set as:
  (3) $$\begin{array}{c} f_{\text{ref}}=f_{\text{main}}\pm \Delta f, \end{array}$$
 where *f*_ref_ is the storage system's reference frequency,  *f*_main_ is the main grid's frequency, and Δ*f* is the minor adjustment frequency, which can be set to 0.05 Hz;
 the selection of addition or subtraction depends on the initial phase angle difference, which can be set as subtraction;
 (c) synchronization check: mode controller makes checking synchronization feature to take effect; when the voltage difference and phase angle difference meet synchronization requests, the mode controller sends a close PCC breaker command;
 (d) after the close command is received, the PCC break turns on, and microgrid operate under grid-connected mode; storage system detects this and works in PQ control mode; planned conversion is completed.

## 3. Simulation Analysis

### 3.1. Simulation Parameters

**Figure 5:**
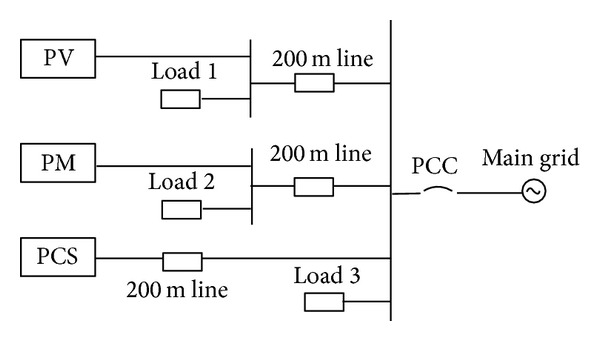
Schematic diagram of the microgrid.

The simulation system is shown in Figure 5. Microgrid has three distributed sources, photovoltaic “PV,” direct-drive wind power “PM,” and battery energy storage system “PCS,” and three groups of loads which are voltage-sensitive.

The photovoltaic generation “PV” works in PQ control mode, where the rated power factor is 1.0, the maximum active power track generation is adopted, and the rated active power is 150 kW. Direct-drive wind power “PM” works in PQ control, where the rated active power is 200 kW, the rated power factor is 1.0, and the maximum active power track is adopted.

In this example, all lines are 380 V line, *R* = 0.642 *Ω*/km, *X* = 0.102 *Ω*/km. Load is constant impedance load, expressed as *Z*_*ld*_ = *R*_*ld*_ + *jX*_*ld*_. Load parameters are as follows:

(4) $$\begin{array}{c} Z_{ld1}=0.922+0.218j\;\Omega ,\\ Z_{ld2}=1.296+0.466j\;\Omega ,\\ Z_{ld3}=1.294+0.466j\;\Omega . \end{array}$$

### 3.2. Simulation Results

#### 3.2.1. Planned Conversion from Grid-Connected Mode to Islanding

**Figure 6:**
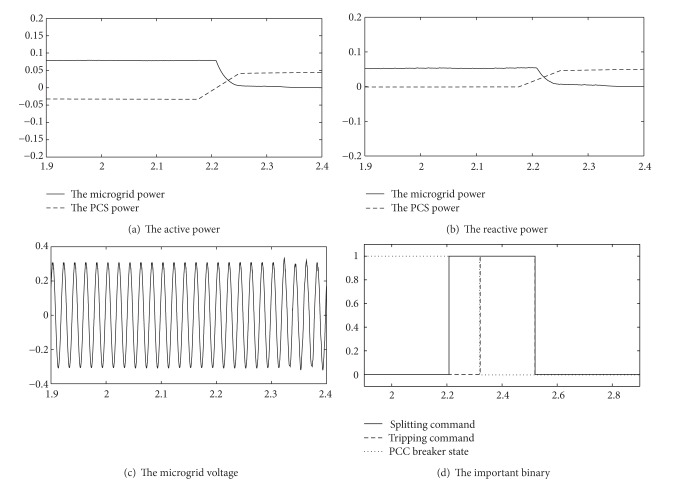
Operation results (1).

Figure 6 shows the operation results.

The microgrid works under the grid-connected mode before 2.2 seconds, and the switching reactive power is 50 kVA; the switching active power is 80 kW.

At 2.2 seconds, the mode controller received the splitting commands. The mode controller began to change the reference power of the storage system until the apparent power reached 0, as shown in Figures 6(a) and 6(b).

After adjustment for 150 milliseconds, the amplitude of apparent power is continuously lower than the threshold value for a period of time, and it meets the tripping conditions; the mode controller of the microgrid sends the command to trip the PCC breaker, as shown in Figure 6(d).

Before and after splitting, the voltage of the microgrid remained unchanged, as shown in Figure 6(c).

Through the simulation results, the mode controller of the microgrid is useful in this conversion and maintains stability of power system voltage before and after splitting.

#### 3.2.2. Unplanned Conversion from Grid-Connected Mode to Islanding

**Figure 7:**
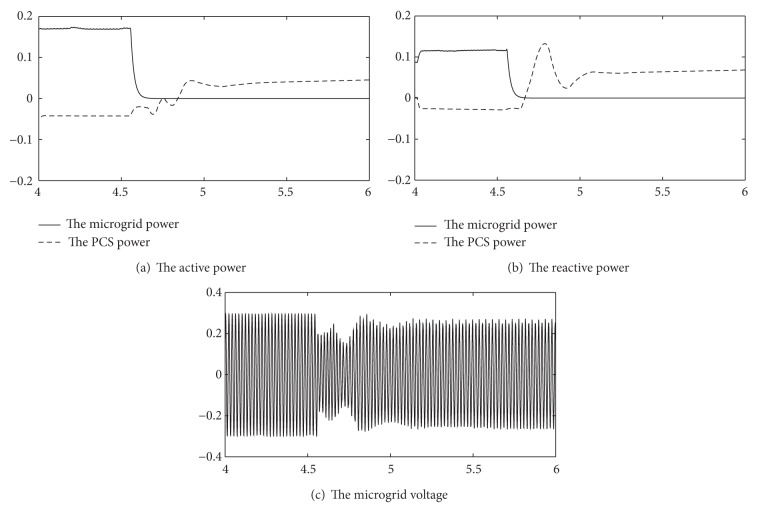
Operation results (2).

Operation results are shown in Figure 7.

The microgrid works under grid-connected mode before 4.5 seconds, and the switching reactive power is 110 kVA; the switching active power is 170 kW.

At 4.5 seconds, the microgrid disconnected from the main grid.

After 200 milliseconds, storage system detected islanding mode, operated under V/F control mode, and adjusted the output power.

At moment of splitting, the voltage will have some changes. In this case, the voltage dropped a little. After the storage system works from PQ control mode to VF control model, the voltage of the microgrid gets gradually regeneration and ultimately achieves stable operation, as shown in Figure 7(c).

Through the simulation results, the storage system control logic is useful in this conversion and maintains stability of power system voltage before and after splitting.

#### 3.2.3. Planned Conversion from Islanding Mode to Grid-Connected Mode

**Figure 8:**
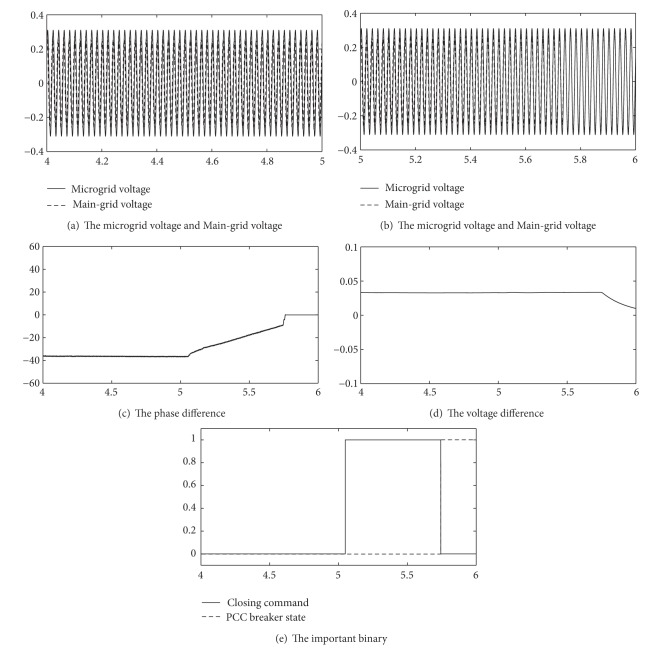
Operation results (3).

Operation results are shown in Figure 8.

The microgrid operates in islanding mode before 5.0 seconds.

The microgrid sends the reclosing command at 5.0 seconds. The voltage of microgrid and the main grid is shown in Figure 8(a). In Figure 8(a), the phase difference between microgrid and main grid is about 40°.

After adjustment about 0.6 seconds, synchronization condition is met at 5.7 seconds, as shown in Figure 8(b). After the mode controller sends a close command, complete the conversion, as shown in Figure 8(e). Figures 8(c) and 8(d) are phase difference and amplitude difference in the process.

The simulation results show that the mode controller of microgrid is valid for synchronous reclosing.

Asynchronous reclosing is not detailed in this paper.

## 4. The Implementation of Device

The operation and control device is the important device of the microgrid, and the mode controller function is one of the important functions in the operation and control device. The characteristic of the operation and control device can influence the stability of the microgrid.

### 4.1. The Architecture of the Operation and Control Device

**Figure 9:**
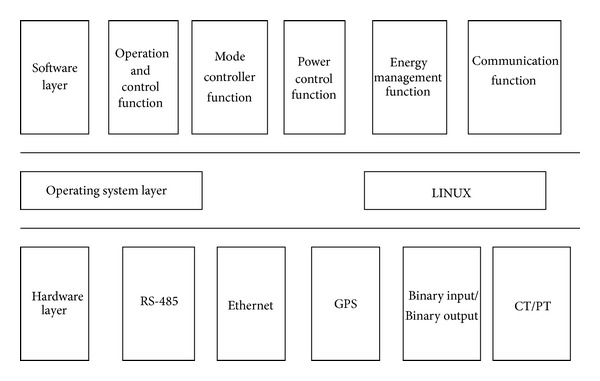
The operation and control device of microgrid.

The architecture of the operation and control device is as shown in Figure 9.

The operation and control device included three layers as follows:

1. hardware layer which includes CPU (Inter D525) and the necessary interface, such as RS-485, Ethernet, GPS, binary input, and binary output,
2. operating system layer which is the operation and control device used LINUX operating system,
3. software layer which includes operation and control function, mode controller function, power control function, energy management function, communication function, and so forth.

The core control strategies of the operation and control device including:

1. microgrid black start,
2. microgrid power control,
3. microgrid optimization control,
4. microgrid mode controller,
5. microgrid energy management.

In order to adapt to different grid structures, different micropower types, multiterminals, and other different applications, the operation and control device needed be programmed by xml file, and then it is consistent with the energy storage device and the secondary control equipment to achieve and mode conversion smoothly.

### 4.2. The Communication of the Operation and Control Device

**Figure 10:**
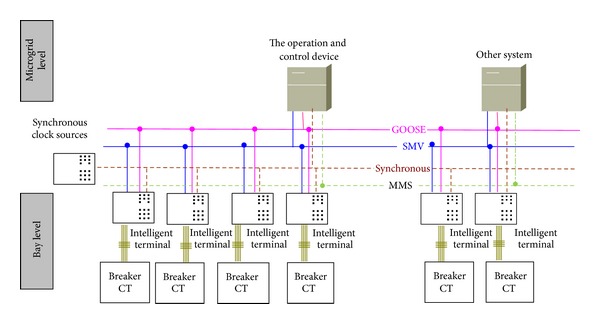
Communication architecture of the microgrid based on IEC61850.

With the purpose of implementing control function, the device needs to communicate with the intelligent terminal in microgrid. The IEC61850 protocol, such as 9-2, GOOSE, and MMS, can be support by purposed device. The device adopts the 61850 communication architecture, and uses the SMV protocol and GOOSE protocol. The communication of microgrid can adopt the networking mode or the point-to-point mode between intelligent terminal and the operation and control device. The communication of the microgrid involves three kinds of network services, which are SMV, GOOSE, and time synchronization network, each of which can use a separate network, or share a common network. In practical applications, the IEEE 1588 Synchronous Ethernet technologies are adopted to build a shared network for SMV, GOOSE, and time synchronization, and optimized data flow distribution is achieved by using VLAN. A typical communication architecture of the microgrid based on IEC61850 is shown in Figure 10.

As shown in Figure 10, the operation and control system of microgrid mainly consists of the following:

1. the operation and control device: each microgrid sets up one operation and control device,
2. intelligent terminal: the intelligent terminal is mainly responsible for collecting AC quantities and acquiring information from local circuit breakers and executing the control commands issued from the central unit,
3. synchronization clock source: the synchronization clock source mainly provides clock synchronization of the intelligent terminal.

## 5. Conclusions

Microgrid operates under two typical modes. Microgrid mode conversion has been an important part of microgrid research, and the seamless transfer of the microgrid is the control goal. The “master-slave” architecture microgrid which is widely used in engineering is selected as the research focus. The mode conversion function is fulfilled by the mode controller of microgrid and the inverter of the energy storage system. The simulation results show that the mode controller and energy storage system inverter operation mode conversion logic is valid. For further research, characteristic analysis of the equipment in the microgrid can make the microgrid more standardized and more reasonable.
